# Renalase in Haemodialysis Patients with Chronic Kidney Disease

**DOI:** 10.3390/jcm10040680

**Published:** 2021-02-10

**Authors:** Magda Wisniewska, Natalia Serwin, Violetta Dziedziejko, Małgorzata Marchelek-Mysliwiec, Barbara Dołegowska, Leszek Domanski, Kazimierz Ciechanowski, Krzysztof Safranow, Andrzej Pawlik

**Affiliations:** 1 Clinical Department of Nephrology, Transplantology and Internal Medicine, Pomeranian Medical University, 70-111 Szczecin, Poland; mwisniewska35@gmail.com (M.W.); malgorzata.marchelek@gmail.com (M.M.-M.); domanle@pum.edu.pl (L.D.); kazcie@pum.edu.pl (K.C.); 2 Department of Microbiology, Immunology and Laboratory Medicine, Pomeranian Medical University, 70-111 Szczecin, Poland; natmat@pum.edu.pl (N.S.); Barbara.Dolegowska@pum.edu.pl (B.D.); 3 Department of Biochemistry and Medical Chemistry, Pomeranian Medical University, 70-111 Szczecin, Poland; viola@pum.edu.pl (V.D.); chrissaf@mp.pl (K.S.); 4 Department of Physiology, Pomeranian Medical University, 70-111 Szczecin, Poland

**Keywords:** renalase, chronic kidney disease, haemodialysis

## Abstract

Chronic kidney disease (CKD) is an inflammatory disease leading to kidney insufficiency and uremia. Renalase is a novel flavoprotein with enzymatic activities. Previous studies have shown that chronic kidney disease may influence renalase serum levels. Renalase metabolises catecholamines and therefore may be involved in the pathogenesis of hypertension and other diseases of the circulatory system. In this study, we examined renalase levels in serum, erythrocytes and urine from haemodialysis CKD patients. The study enrolled 77 haemodialysis CKD patients and 30 healthy subjects with normal kidney function as the control group. Renalase serum and urine concentrations in CKD patients were significantly increased when compared with control subjects (185.5 ± 64.3 vs. 19.6 ± 5.0 ng/mL; *p* < 0.00001 and 207.1 ± 60.5 vs. 141.6 ± 41.3 ng/mL; *p* = 0.00040, respectively). In contrast, renalase levels in erythrocytes were significantly lower in CKD patients when compared with control subjects (176.5 ± 60.9 vs. 233.2 ± 83.1 ng/mL; *p* = 0.00096). Plasma levels of dopamine, adrenaline and noradrenaline were also significantly lower in CKD patients when compared with controls. Conclusions: Increased serum and urine concentrations of renalase in haemodialysis CKD patients are likely related to compensatory production in extrarenal organs as a result of changes in the cardiovascular system and hypertension. The decreased plasma concentrations of catecholamines may be due to their increased degradation by plasma renalase. Decreased renalase levels in erythrocytes may be probably due to lower renalase synthesis by the kidneys in CKD. The results indicate the presence of renalase in erythrocytes.

## 1. Introduction

Chronic kidney disease (CKD) is an inflammatory disease leading to kidney insufficiency and uremia [1]. Many metabolic changes have been found in this disease that increase the risk of developing cardiovascular complications. Numerous enzymes, cytokines and other mediators are involved in the development of circulatory system diseases in patients with CKD [2,3].

Renalase is a flavoprotein with enzymatic activities of amine oxidase detected in 2005 by Xu et al. [4]. This enzyme is mainly released in proximal tubules of the kidney but also by hepatocytes, cardiomyocytes and myocytes in skeletal muscles. Renalase is also produced in adrenal cells, adipose tissue and the central and peripheral nervous system [5]. Renalase metabolises catecholamines and therefore may be involved in the pathogenesis of hypertension and other diseases of the circulatory system [6,7,8]. Therefore, some authors suggest that this enzyme may be the risk factor for developing circulatory system diseases. Previous studies showed that chronic kidney disease (CKD) may influence renalase serum levels [6]; however, the results are inconsistent. The results of some studies suggest that patients with CKD have lower levels of renalase in serum, while others suggest that CKD patients have increased levels of renalase [1,8,9,10]. In the previous work, we studied renalase concentrations in CKD patients with preserved renal function and correlated with parameters of kidney function [11]. Patients with end-stage kidney disease most often suffer from anuria and require hemodialysis, which changes many biochemical parameters. The aim of this study was to examine renalase levels in serum, erythrocytes and urine from haemodialysis CKD patients.

## 2. Materials and Methods

### 2.1. Patients

This study included 77 patients with CKD. The causes of CKD were hypertension (33.8%), diabetes mellitus (22.1%), glomerular kidney disease (15.6%), polycystic kidney disease (6.5%), birth defects (3.9%) and unknown (18.1%).

The control group included 30 healthy subjects, with normal kidney function (normal GFR values and normal creatinine serum levels) [11]. The GFR was estimated using the CKD-EPI equation (eGFR). All participants from the study group were patients in the Clinic of Nephrology, Transplantology and Internal Diseases, Pomeranian Medical University in Szczecin, Poland. The study was approved by the ethics committee in Pomeranian Medical University, Szczecin, Poland (KB-0012/122/14), and written informed consent was obtained from all subjects.

### 2.2. Methods

Venous blood sampling was prepared in the morning with the use of Sarstedt Monovette tubes (Nümbrecht, Germany) containing a clotting activator. Serum samples were prepared by centrifugation (10 min, 1000× *g*) and stored frozen until use.

Additionally, whole blood samples were drawn into K_3_EDTA Monovette tubes (Sarstedt, Nümbrecht, Germany) to obtain erythrocyte lysates. Erythrocytes were separated from plasma by centrifugation (10 min, 1000× *g*) and washed three times saline (0.9%). Each wash was followed by centrifugation as described above and removal of the supernatant fraction. Erythrocytes samples were stored frozen at −80 °C until use. Before use, to obtain erythrocytes lysis, samples were diluted 1:3 (*v*/*v*) in distilled water. The dilution coefficient was included in the determination of haemoglobin concentration measured with the use of Drabkin’s reagent (Sigma-Aldrich, St Louis, MO, USA) Renalase in erythrocytes was measured using a serum renalase kit (WuHan EIAab, Wuhan, China).

Serum, erythrocytes and urine concentration of the renalase were assessed with the use of the human renalase specific ELISA kit (WuHan EIAab, Wuhan, China). Additionally, adrenaline, noradrenaline and dopamine concentrations were evaluated in blood plasma with the use of the ELISA kit (LDN Labour Diagnostika Nord GmbH & Co. KG, Nordhorn, Germany). The total concentration of renalase in the erythrocytes (expressed in ng/mL), as well as the concentration of renalase expressed per 1 g of Hb (ng/1 g Hb), are presented. Renalase concentration is expressed per 1 g of Hb to account for variability in the erythrocyte and haemoglobin values of the patients.

Standard laboratory serum parameters were determined using the Architect c8000 analyser (ABBOTT, Austin, TX, USA).

### 2.3. Statistical Analysis

Data were analysed with STASTISTICA 12.5 program (StatSoft, Tulsa, OK, USA). Shapiro–Wilk test was used to verify normality of distributions. It showed that most quantitative variables have distributions significantly different from normal distribution. Therefore, non-parametric Mann–Whitney test was used to compare values between groups. The strength of correlations between selected quantitative parameters was measured using non-parametric Spearman’s rank correlation coefficient (R_S_). Associations with *p* < 0.05 were considered statistically significant for all analyses.

## 3. Results

In this study, we measured renalase levels in serum, erythrocytes and urine, as well as plasma concentrations of dopamine, noradrenaline and adrenaline, in healthy subjects and haemodialysis CKD patients. Serum and urine renalase levels in CKD patients were significantly increased when compared with control subjects (185.5 ± 64.3 vs. 19.6 ± 5.0 ng/mL; *p* < 0.00001 and 207.1 ± 60.5 vs. 141.6 ± 41.3 ng/mL; *p* = 0.0004, respectively). However, renalase levels in erythrocytes were significantly lower in CKD patients when compared with control subjects (176.5 ± 60.9 vs. 233.2 ± 83.1 ng/mL; *p* = 0.00096) (Table 1). Plasma levels of adrenaline, noradrenaline and dopamine were also significantly lower in CKD patients when compared with controls (Table 1).

**Figure 1:**
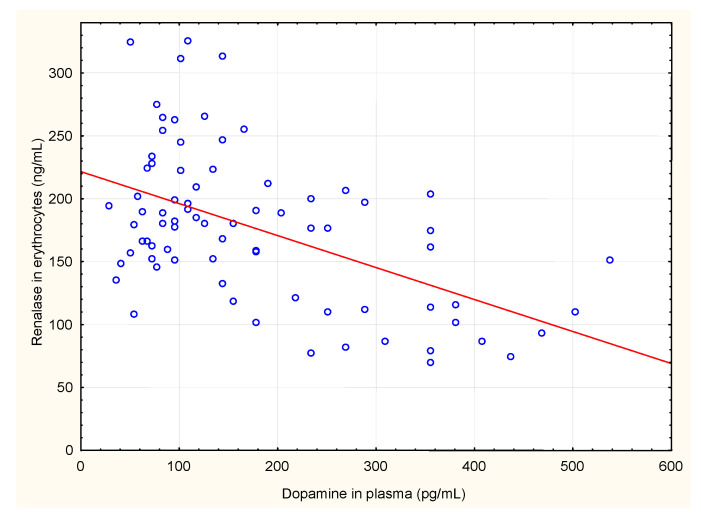
Correlation between concentrations of renalase in erythrocytes and dopamine in plasma of haemodialysis patients.

**Figure 2:**
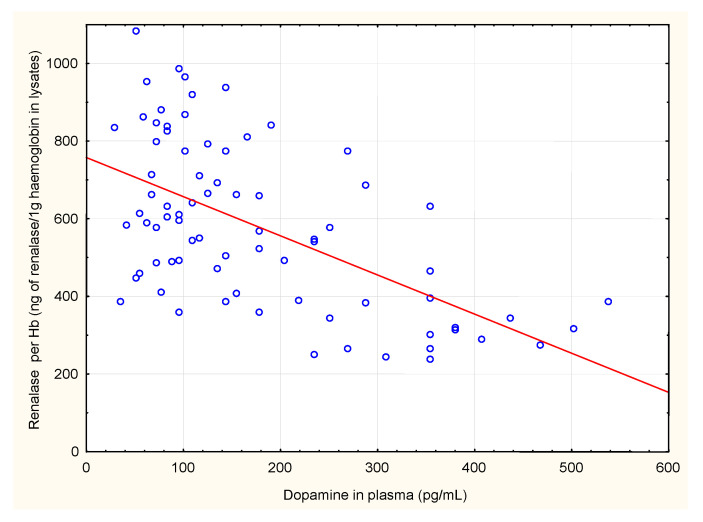
Correlation between concentrations of renalase per Hb (ng of renalase/1 g haemoglobin in lysates) and dopamine in plasma of hemodialysis patients.

**Figure 3:**
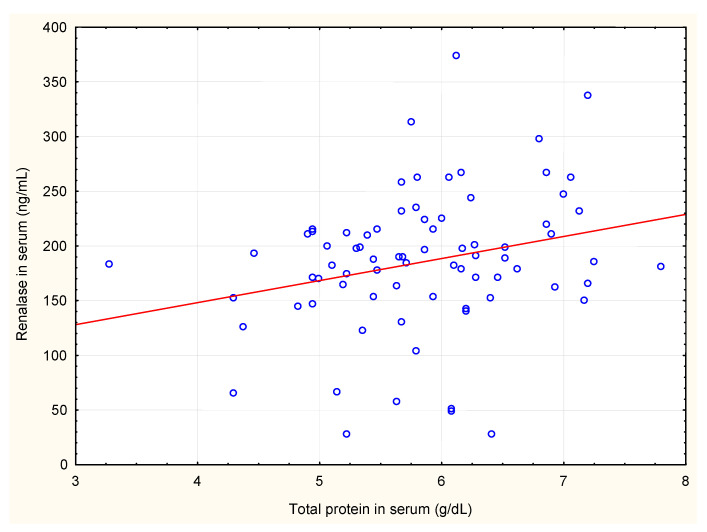
Correlation between serum concentrations of renalase and total protein in haemodialysis patients.

**Figure 4:**
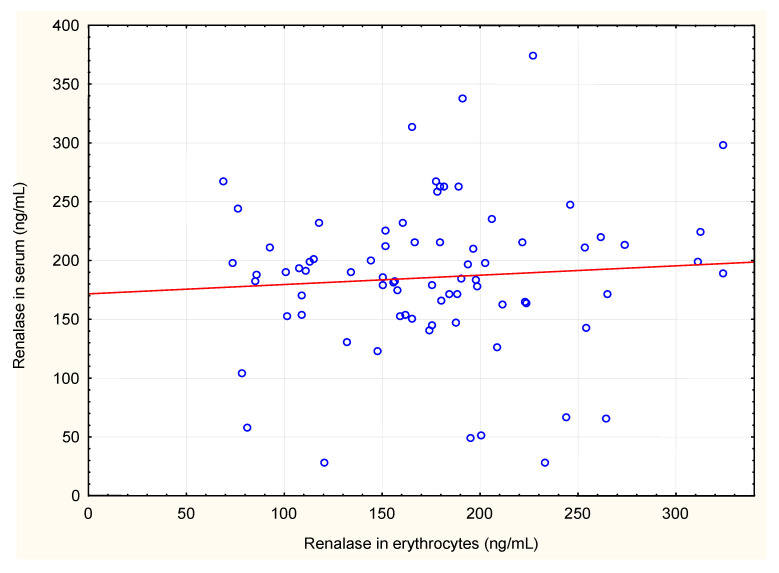
Correlation between concentrations of renalase in serum and erythrocytes of haemodialysis patients.

We also analysed correlations between renalase levels in serum and in erythrocytes and renalase levels, calculated per gram of haemoglobin, with plasma levels of adrenaline, noradrenaline, dopamine, serum total protein, serum albumin and haemodialysis diuration. Renalase levels in erythrocytes, and renalase levels calculated per gram of haemoglobin correlated negatively with plasma dopamine concentrations. Renalase serum levels positively but weakly correlated with serum total protein (Table 2, Figure 1, Figure 2 and Figure 3). There were no significant correlations between serum and erythrocyte renalase concentrations, in both hemodialysis patients and controls (Figure 4).

## 4. Discussion

The aim of this study was to examine renalase levels in serum, erythrocytes and urine in haemodialysis CKD patients. To our knowledge, this is the first study examining the concentrations of renalase in hemodialysis CKD patient erythrocytes. Our results showed that serum and urine renalase levels in CKD patients were significantly increased, whereas erythrocyte levels were lower when compared to healthy patients. Erythrocytes, being nuclear-free cells without mitochondria, do not carry out classical metabolism and, therefore, are a very good “transporter” of many substances. Previous studies have shown that the concentration of renalase is significantly increased in patients with chronic kidney disease (CKD), and additional studies have demonstrated that this change is correlated with disease severity [8,9,10,11]. Patients with CKD characteristically display a high level of anaemia that is mainly associated with impaired erythropoietin production, although haemolysis and a significantly shortened erythrocyte survival time have also been implicated [12,13]. This phenomenon is even more common in hemodialysis (HD) patients. Additionally, erythrocytes are involved in the storage of many cytokines involved in cell signalling [14]. Renalase, as a potential cytokine, could also be subject to such transport and storage. Moreover, the receptor for renalase has been shown to be PMCA4b, one of the four isoforms of the PMCA calcium pump. PMCA4 is the main isoform of this pump in erythrocytes [15]. Therefore, it cannot be ruled out that renalase without the involvement in catecholamine metabolism is responsible for autocrine functions. Renalase also exhibits enzymatic activity, although only at the intracellular level, stabilizing the active NAD(P)H isoform. Erythrocytes are a reservoir of a very large number of enzymes, primarily antioxidants, which provide erythrocytes with access to ATP and NAD(P)H [16]. Many of these enzymes require NADPH, which is derived from the pentose phosphate pathway. Our results indicate that renalase is present in erythrocytes.; however, the function of renalase in erythrocytes is not yet known.

Previous studies have shown that renalase levels in CKD patients were decreased, suggesting correlations between lower serum renalase levels and increased catecholamine levels. Xu et al. indicated that patients with end-stage renal disease had decreased plasma levels of renalase when compared with healthy subjects [4]. In another study, Desir et al. indicated that increased catecholamine levels in CKD cause renalase deficiency and contribute to hypertension and cardiovascular disease [5,6].

In contrast, studies by Małyszko et al. suggested that CKD patients and kidney allograft recipients had increased plasma renalase levels [8,9,10]. Zbroch et al. examined serum renalase levels in haemodialysis patients [9]. Mean serum renalase levels in patients were significantly higher than in the control group. Moreover, serum renalase levels were significantly lower in patients with residual renal functions when compared to anuric patients. There was a significant inverse correlation between serum renalase levels and residual renal function. In another study, renalase levels were higher in dialysis groups when compared to healthy subjects [10]. Renalase levels correlated with dialysis vintage, and inversely correlated with residual diuresis. Elevated circulating renalase levels in dialysis patients may be related to kidney function and activation of the sympathetic nervous system detected in these patients.

Furthermore, other studies have suggested that renalase levels were positively correlated with impaired kidney function [17,18]. Stojanovic et al. indicated that renalase levels were negatively correlated with estimated glomerular filtration rates and positively correlated with creatinine levels [19]. Baek et al. suggest that increased levels of renalase in CKD patients may be the risk factor of several cardiovascular complications [20].

Previous studies have indicated that renalase not only has enzymatic properties, but also should be considered as a cytokine [21]. Additionally, renalase may exert anti-apoptotic action and may protect against ischemia reperfusion injury and toxicity induced by cisplatin [22]. It has been shown that renalase may activate several signalling pathways associated with of extra-cellular signal-regulated kinase, protein kinase B and p38 mitogen-activated kinase [23]. Additionally, renalase protects against ischemic acute kidney injury and renal fibrosis via inhibiting oxidative [24,25,26].

Li et al. have indicated increased renalase serum levels in CKD patients, which correlated with increased levels of endotelin, a hormone involved in pathogenesis of cardiovascular diseases [27].

The expression of renalase is regulated by dopamine receptors, especially receptor D5. It is likely that dysfunction of this receptor and aberrant regulation of renalase function may be associated with development of hypertension [28].

In our previous study, we examined renalase concentrations in CKD patients with preserved renal function [11]. Renalase serum concentrations correlated negatively with parameters of kidney function. These results suggest that renalase concentrations increase with the development of the disease and the deterioration of renal function.

In this study, we examined renalase levels in haemodialysis patients with CKD. These patients had increased levels of renalase in the serum and urine and lower levels in erythrocytes. The studies indicate that renalase production is primarily impaired in CKD patients and increases with disease development. Increased serum and urine concentrations of renalase in haemodialysis CKD patients are likely related to compensatory production in extrarenal organs as a result of changes in the cardiovascular system and hypertension. The decreased plasma concentrations of catecholamines may be due to their increased degradation by serum renalase. This may confirm the role of renalase in catecholamine metabolism. We hypothesise that decreased renalase levels in CKD patient erythrocytes may be due to primarily lower renalase synthesis by the kidneys in CKD. Nevertheless, renalase’s function and properties remain poorly understood and require further investigation.

## Figures and Tables

**Table 1:** Comparison of studied parameters between hemodialysis patients and control subjects.

Parameters	Control		HD Patients		HD vs. Control
	Mean ± SD	Median (Q1–Q3)	Mean ± SD	Median (Q1–Q3)	*p* ^&^
Age (years)	57.37 ± 18.46	54.5 (45.0–72.0)	65.44 ± 15.55	68.0 (57.0–77.0)	0.027
HGB (mmol/L)	7.89 ± 0.63	7.95 (7.30–8.40)	6.77 ± 1.00	6.90 (6.10–7.40)	<0.00001
RBC (T/L)	4.85 ± 0.41	4.84 (4.50–5.21)	3.54 ± 0.53	3.50 (3.19–3.90)	<0.00001
MCHC (mmol/L)	19.48 ± 0.65	19.5 (18.9–19.9)	20.54 ± 0.61	20.6 (20.2–21.0)	<0.00001
Uric acid in serum (mg/dL)	5.51 ± 0.61	5.55 (5.00–6.00)	7.42 ± 1.83	7.21 (6.40–8.20)	<0.00001
Total protein in serum (g/dL)	7.44 ± 0.43	7.45 (7.30–7.80)	5.85 ± 0.82	5.80 (5.31–6.28)	<0.00001
Albumin in serum (g/dL)	3.70 ± 0.16	3.75 (3.60–3.80)	3.18 ± 0.51	3.25 (2.89–3.55)	<0.00001
Glucose in serum (mg/dL)	86.67 ± 7.19	87.0 (81.0–92.0)	108.55 ± 36.14	98.0 (86.0–125)	0.00096
Creatinine in serum (mg/dL)	0.83 ± 0.12	0.82 (0.74–0.94)	8.42 ± 3.58	8.40 (5.75–10.3)	<0.00001
Creatinine in urine (mg/dL)	90.03 ± 54.06	78.4 (42.5–125)	90.76 ± 77.55	65.0 (40.0–124)	0.64
Adrenaline in plasma (pg/mL)	30.45 ± 33.88	17.7 (10.5–39.3)	14.64 ± 11.81	12.2 (7.32–18.3)	0.016
Noradrenaline in plasma (pg/mL)	399.10 ± 318.99	275 (197–604)	345.74 ± 999.82	118 (51.1–228)	0.00019
Dopamine in plasma (pg/mL)	440.95 ± 343.22	313 (215–638)	177.26 ± 124.40	135 (83.3–252)	<0.00001
Renalase in serum (ng/mL)	19.63 ± 4.99	17.7 (16.3–21.8)	185.55 ± 64.31	187 (154–215)	<0.00001
Renalase in erythrocytes (ng/mL)	233.23 ± 83.11	254 (166–293)	176.51 ± 60.93	178 (135–206)	0.00096
Renalase Hb (ng/1 g Hb)	697.65 ± 273.43	707 (485–857)	579.11 ± 214.26	575 (387–772)	0.04
Renalase in urine (ng/mL)	141.57 ± 41.31	144 (116–170)	207.14 ± 60.53	205 (150–260)	0.0004
Renalase/creatinine (ng/1 mg creatinine in urine)	252.38 ± 210.81	201 (96.7–285)	431.01 ± 333.13	354 (179–480)	0.031

^&^ Mann–Whitney U test. HD patients—hemodialysis patients; HGB—haemoglobin; MCHC—mean corpuscular haemoglobin concentration; Q1—lower quartile; Q3—upper quartile; Renalase Hb—renalase concentrations calculated as hemoglobin (ng of renalase/1 g haemoglobin in lysates); RBC—red blood cells; SD—standard deviation.

**Table 2:** Correlations between studied parameters in hemodialysis patients.

Parameters	Renalase in Serum		Renalase in Erythrocytes		Renalase Hb	
	R_s_	*p*	R_s_	*p*	R_s_	*p*
Adrenaline in plasma	0.07	0.56	0.06	0.62	−0.04	0.73
Noradrenaline in plasma	0.20	0.085	0.06	0.61	0.11	0.35
Dopamine in plasma	−0.08	0.51	−0.42	0.00013	−0.55	<0.00001
Serum total protein	0.24	0.034	−0.05	0.67	−0.05	0.68
Serum albumin	−0.10	0.39	0.00	0.97	−0.08	0.48
Duration of hemodialysis	0.08	0.50	0.02	0.83	−0.02	0.84

R_s_—Spearman rank correlation coefficient. Hb renalase concentrations calculated as haemoglobin (ng of renalase/1 g haemoglobin in lysates).

## Data Availability

Data sharing not applicable.
